# From Diet to Oral and Periodontal Health: Exploring the Crucial Role of Nutrition—A Narrative Review

**DOI:** 10.3390/nu18010168

**Published:** 2026-01-05

**Authors:** Florin Razvan Curca, Ionut Luchian, Florinel Cosmin Bida, Dragos Ioan Virvescu, Gabriel Rotundu, Oana Maria Butnaru, Gheorghe Balan, Zinovia Surlari, Andrei Georgescu, Liliana Pasarin, Dana Gabriela Budala

**Affiliations:** Grigore T. Popa University of Medicine and Pharmacy, 700115 Iasi, Romania

**Keywords:** nutrition, oral health, diet, oral cavity, periodontal disease

## Abstract

**Background:** The growing body of evidence linking dietary factors to oral and periodontal health is characterized by substantial heterogeneity in study design, dietary assessment methods, and reported outcomes, warranting a comprehensive narrative synthesis. Diet is a key determinant of oral and periodontal health, influencing inflammation, oxidative stress, salivary composition, and the oral microbiome. **Objectives**: This narrative review aims to synthesize current clinical, epidemiological, and mechanistic evidence on how dietary patterns and specific nutrients affect oral and periodontal health, focusing on inflammatory pathways, microbiome modulation, nutrient-dependent tissue mechanisms, and clinical outcomes. **Methods**: A structured narrative search was conducted in PubMed, Scopus, Web of Science, and Google Scholar (2000–2025). Studies examining diet, nutrients, the oral microbiome, caries, gingival inflammation, or periodontal disease were screened through a multistep process, resulting in 98 included articles. **Results**: High-sugar and ultra-processed diets trigger inflammation and oral dysbiosis, increasing caries and periodontal susceptibility. In contrast, nutrient-rich and anti-inflammatory diets improve immune regulation, support microbial balance, and are associated with better periodontal parameters. **Conclusions**: Dietary habits significantly shape oral and periodontal outcomes through interconnected metabolic, microbial, and immunological pathways. Integrating targeted nutritional counseling into dental care may strengthen prevention strategies and improve long-term oral health.

## 1. Introduction

Diet plays a central and often underestimated role in shaping oral and periodontal health. Diet, in addition to its well-known nutritional benefits, significantly impacts the oral ecology [1]. This influence extends to the composition of saliva, its ability to neutralize acids, the diversity of oral microbes, the body’s inflammatory reactions, and the health of teeth and gums [2,3]. In recent decades, an increasing body of research has underscored the interconnectedness of oral health and systemic nutrition. Dietary habits and the availability of nutrients significantly influence immune function, oxidative stress, metabolic balance, and the host’s capacity to combat periodontal pathogens [4,5].

Dietary patterns and nutrient intake have been increasingly associated with oral and periodontal health outcomes, including caries development, gingival inflammation, and periodontal disease progression. Observational and interventional studies suggest that dietary quality, sugar intake, and micronutrient status may influence oral health through metabolic, inflammatory, and microbial pathways [3,4,5].

Global shifts toward Westernized eating habits—characterized by high intakes of sugars, refined carbohydrates, saturated fats, and ultra-processed foods, have contributed to rising rates of dental caries, gingivitis, and periodontitis [6]. Periodontal disease is a chronic inflammatory condition influenced by both local and systemic factors. Nutritional exposures have been implicated in modulating inflammatory responses relevant to periodontal tissue breakdown, positioning diet as a potential modifier of disease susceptibility and progression [6,7].

Frequent sugar consumption and fermentable carbohydrates promote acidogenic shifts in the oral biofilm, driving demineralization and caries development, while nutrient-poor, pro-inflammatory diets amplify gingival and periodontal inflammation [7]. At the same time, deficiencies in vitamins C and D, omega-3 fatty acids, calcium, magnesium, and antioxidants impair wound healing, immune regulation, collagen stability, and alveolar bone metabolism, increasing susceptibility to periodontal breakdown [8,9,10,11].

Conversely, anti-inflammatory dietary patterns such as the Mediterranean diet, DASH diet, and plant-forward diets have been associated with reduced periodontal inflammation, lower oxidative stress, improved microbial diversity, and enhanced clinical outcomes following periodontal therapy [12,13,14]. An anti-inflammatory diet refers to a dietary pattern characterized by a high intake of fruits, vegetables, whole grains, legumes, nuts, and unsaturated fatty acids, alongside a reduced consumption of refined carbohydrates, saturated fats, and ultra-processed foods. This pattern is generally associated with lower inflammatory potential. This effect is attributed to its high nutrient density and the presence of bioactive compounds with antioxidant and immunomodulatory properties.

The oral microbiome represents a key interface between dietary factors and periodontal health. Emerging evidence indicates that dietary composition may influence microbial diversity and metabolic activity, thereby affecting host–microbe interactions in the oral cavity [15].

Recent microbiome studies further highlight the diet–oral ecology axis, showing that fiber-rich, polyphenol-rich, and omega-3–rich diets foster a more eubiotic microbiome, whereas high-sugar and high-fat diets promote dysbiosis and overgrowth of pathogenic species [15,16].

Given the strong bidirectional relationship between diet, systemic inflammation, metabolic disease, and oral health, a comprehensive understanding of nutritional influences is essential for modern dental practice.

Despite growing interest in diet–periodontal interactions, the available evidence remains heterogeneous with respect to study design, dietary assessment methods, and reported outcomes. This variability supports the use of a narrative review approach to integrate findings across observational, interventional, and mechanistic studies. While periodontal disease provides the principal framework for mechanistic discussion, dietary influences on dental caries and broader oral health outcomes are also considered to provide a comprehensive oral health perspective.

Therefore, this narrative review aims to comprehensively synthesize current clinical, epidemiological, microbiome-focused, and mechanistic evidence on how dietary patterns and specific nutrients influence oral and periodontal health. It highlights the interconnected inflammatory, microbial, and metabolic pathways through which nutrition shapes disease susceptibility and clinical outcomes.

Throughout this review, associations between dietary factors and oral or periodontal outcomes are described using language intended to reflect correlation rather than causation. Given the narrative design of the review and the heterogeneity of study designs, populations, and assessment methods, terms such as “associated with,” “linked to,” or “may contribute to” are used to avoid overstating the strength of the evidence. Reported relationships should therefore be interpreted as indicative of potential associations rather than definitive causal effects.

Interpretation of existing literature must also consider the inherent limitations of dietary assessment methodologies. Many observational studies rely on self-reported dietary instruments, particularly food frequency questionnaires, which are subject to recall bias, misreporting, and limited sensitivity to short-term dietary changes. These tools may inadequately capture portion sizes, food preparation methods, and complex dietary patterns, potentially leading to exposure misclassification. As a result, variability in dietary assessment represents a critical factor influencing the strength and consistency of reported associations between diet and oral or periodontal outcomes.

## 2. Literature Review

Given the complexity of nutritional interactions with biological, microbial, and behavioral pathways, a narrative review format was chosen to allow for the integration of heterogeneous sources, including clinical trials, observational studies, mechanistic research, and established nutritional frameworks. Although not structured as a systematic review, the methodology followed a transparent and predefined approach to ensure rigor and reproducibility in the identification, selection, and synthesis of evidence.

### 2.1. Methodology

#### 2.1.1. Research Questions

The review was guided by a set of predefined research questions intended to frame the scope and ensure a coherent exploration of the diet–oral health relationship. Specifically, the review sought to clarify how dietary patterns and specific nutrients influence oral and periodontal health, which biological and microbiome-mediated mechanisms underpin these associations, and to what extent nutritional factors contribute to caries development, periodontal inflammation, and alterations in the oral microbiome.

Furthermore, the review aimed to identify the protective potential of anti-inflammatory or nutrient-rich diets, as well as the detrimental effects of high-sugar or pro-inflammatory dietary habits. By addressing these guiding questions, the review integrates evidence across clinical, epidemiological, and mechanistic research domains to provide a comprehensive understanding of the role of nutrition in oral and periodontal health.

#### 2.1.2. Search Strategy

The literature search was conducted between January 2000 and January 2025 using four major databases: PubMed/MEDLINE, Scopus, Web of Science, and Google Scholar for supplementary screening. A structured strategy was developed to identify publications investigating the relationship between diet, nutrition, and oral or periodontal health. The search employed combinations of controlled vocabulary and free-text terms, integrating keywords such as “diet,” “nutrition,” “nutrients,” “dietary patterns,” “oral health,” “dental caries,” “periodontitis,” “gingival inflammation,” and “oral microbiome,” connected through Boolean operators. Search strings were adapted to each database to maximize sensitivity while avoiding irrelevant retrieval.

Reference lists of relevant reviews and primary studies were manually screened to identify additional eligible sources not captured during the electronic search. Only peer-reviewed articles published in English were considered. This structured yet flexible search approach ensured comprehensive coverage of both clinical and mechanistic literature relevant to the diet–oral health axis.

#### 2.1.3. Study Selection Process

The study selection process followed a structured multistep approach to ensure transparency and consistency throughout the review. All records identified through database searches were first screened at the title and abstract level to eliminate studies that were clearly irrelevant to the diet–oral health relationship. Articles considered potentially eligible were subsequently retrieved in full text and evaluated in detail, with attention to methodological quality, clarity of dietary assessment, and the presence of oral or periodontal outcomes.

**Figure 1:**
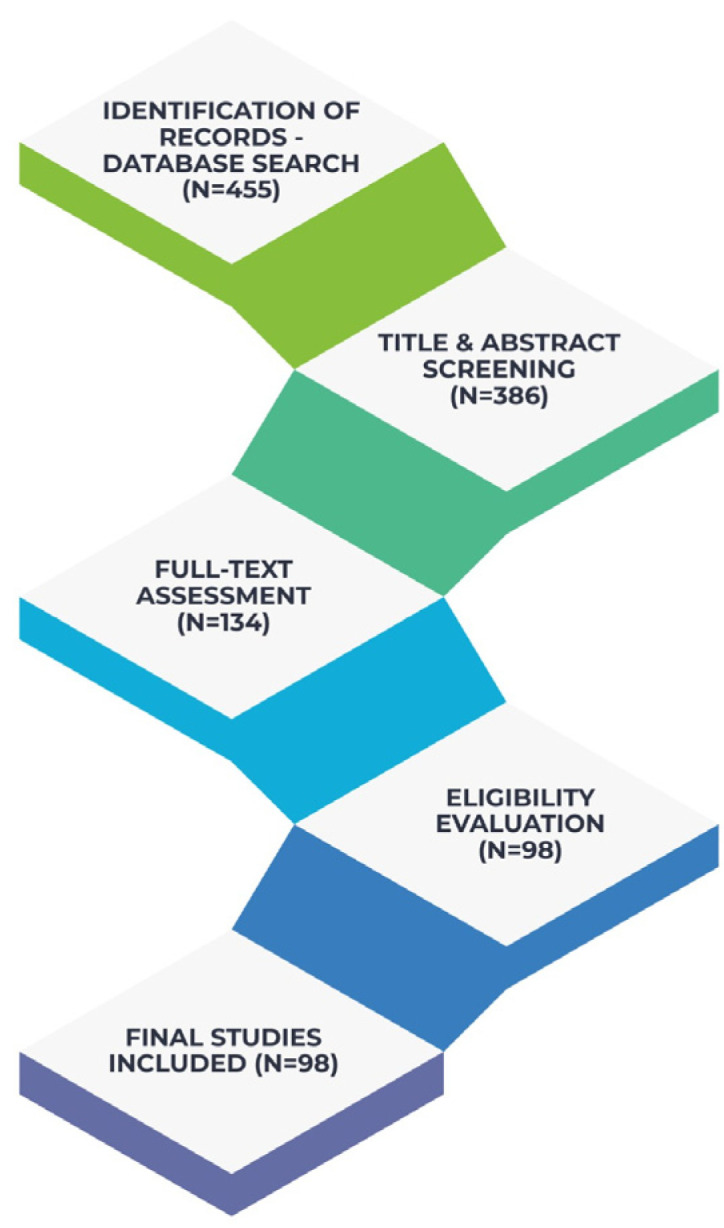
Literature Identification and Study Selection.

Screening was conducted independently by two reviewers, and any disagreements were resolved through discussion until consensus was achieved. From an initial pool of 455 records, duplicates were removed and the remaining studies were assessed through these steps, leading to the inclusion of 98 articles in the final synthesis. The remaining publications were used to inform the overall conceptual framework and background of the narrative synthesis but were not individually cited due to space considerations and overlap in thematic content. Although not accompanied by a PRISMA flow diagram—given the narrative review design, the selection process adhered to a transparent and reproducible methodology as shown in Figure 1, that illustrates the conceptual framework of the study.

This review was conducted as a narrative literature review to allow the integration of heterogeneous evidence derived from clinical, epidemiological, microbiome-focused, and mechanistic studies. Given the diversity of study designs, dietary assessment methods, and oral health outcomes reported in the literature, a systematic review framework was not considered appropriate. Accordingly, this manuscript does not aim to follow PRISMA reporting guidelines or to perform formal risk-of-bias or quality assessments, but rather to provide a comprehensive and structured narrative synthesis of existing evidence.

Although 98 publications were identified as relevant and informed the narrative synthesis, only 83 studies are explicitly cited in the reference list, as conceptually overlapping or background-supporting articles were integrated collectively rather than referenced individually.

#### 2.1.4. Eligibility Criteria

Eligibility criteria were defined as a priori to ensure consistency in the selection of relevant literature. Only studies published in English between 2000 and 2025 that examined the relationship between diet, specific nutrients, or broader dietary patterns and oral or periodontal health outcomes were considered eligible. These outcomes included dental caries, gingival inflammation, periodontitis, salivary biomarkers, and diet-induced alterations of the oral microbiome.

A wide range of study designs was accepted, including randomized controlled trials, observational studies (cohort, case–control, and cross-sectional), systematic reviews and meta-analyses, as well as mechanistic research providing insight into inflammatory, oxidative, or microbial pathways. In vitro and animal studies were included only when they offered mechanistic relevance to human oral physiology.

Studies lacking clear methodological detail, those unrelated to oral outcomes, and non–peer-reviewed materials such as conference abstracts or opinion papers were excluded. Articles focusing solely on systemic nutritional issues without an oral component were also omitted. These criteria ensured the inclusion of high-quality evidence capable of contributing meaningfully to an integrated narrative synthesis.

#### 2.1.5. Data Extraction

Data extraction was performed using a structured approach to ensure consistency across the diverse body of literature included in the review. For each eligible study, relevant information was manually collected by two independent reviewers, focusing on the type of dietary exposure examined, methodological design, characteristics of the study population, and the specific oral or periodontal outcomes measured.

Additional details such as microbiological findings, inflammatory or biochemical markers, nutrient categories, and proposed mechanistic pathways were also recorded when available. Extracted data were compiled into a unified framework to facilitate thematic comparison across studies.

Any discrepancies or uncertainties identified during the extraction process were resolved through discussion and consensus, ensuring the accuracy and reliability of the final dataset.

#### 2.1.6. Data Synthesis

Due to the substantial heterogeneity in study designs, dietary assessment tools, and reported oral health outcomes, a qualitative narrative synthesis was employed to integrate findings across diverse types of evidence. Studies were evaluated and interpreted within broader thematic domains rather than pooled quantitatively.

This approach allowed the integration of clinical, epidemiological, microbiome-focused, and mechanistic research, facilitating a comprehensive understanding of how diet influences oral and periodontal health through inflammatory pathways, oxidative stress, host–microbial interactions, and metabolic regulation.

Evidence was synthesized across major themes, including dietary modulation of systemic inflammatory burden, effects on the oral microbiome, nutrient-specific influences on caries and periodontal inflammation, and the clinical relevance of protective versus harmful dietary patterns. The narrative approach enabled the identification of converging mechanisms and clinical implications, despite methodological variability across studies.

Based on research questions and predefined analytical framework, the studies included were synthesized across several major themes reflecting the principal pathways through which diet influences oral and periodontal health. These themes correspond directly to the mechanisms and outcomes identified as central to the review-systemic inflammatory responses, diet-driven microbial shifts, nutrient-specific biological effects, and clinical manifestations.

In interpreting the mechanistic pathways discussed in this review, it is important to distinguish between biological plausibility and demonstrated clinical effect. While experimental and mechanistic studies provide valuable insights into potential pathways linking diet, inflammation, and the oral microbiome, such evidence does not necessarily equate to clinically confirmed outcomes. In several instances, mechanistic hypotheses are supported primarily by in vitro models, animal studies, or extrapolation from systemic inflammatory research, rather than by oral-specific human intervention studies. These mechanistic frameworks should therefore be viewed as explanatory models that complement, but do not replace, clinical evidence.

### 2.2. Dietary Exposure and Systemic Host Response

Dietary habits exert a profound influence on systemic inflammatory and oxidative pathways, both of which modulate the host response and contribute to susceptibility to oral and periodontal disease. Diets high in refined carbohydrates, saturated fats, and ultra-processed foods promote elevations in circulating inflammatory markers such as IL-6, TNF-α, and *C*-reactive protein, creating a pro-inflammatory systemic environment that exacerbates periodontal tissue breakdown in response to bacterial challenge [17,18]. Such dietary patterns also contribute to postprandial hyperglycemia and the accelerated formation of advanced glycation end products. These processes amplify oxidative stress and impair collagen integrity and wound-healing capacity in periodontal tissues [19].

In contrast, nutrient-dense anti-inflammatory diets—particularly those rich in omega-3 polyunsaturated fatty acids, antioxidants, vitamins C and D, polyphenols, and dietary fiber—reduce systemic inflammatory burden and enhance immune regulation. Omega-3 fatty acids promote the synthesis of specialized pro-resolving mediators (SPMs), which support resolution of inflammation and limit excessive neutrophil activity implicated in periodontal destruction [20]. Antioxidants such as vitamin C, carotenoids, and polyphenols mitigate oxidative damage, support fibroblast function, and contribute to stable collagen turnover, thereby enhancing host resistance to periodontal challenge [21]. Adequate vitamin D status further contributes to improved innate immunity and bone metabolism, effects that have been linked to reduced periodontal inflammation and improved treatment outcomes [22].

Dietary fiber also indirectly influences periodontal health through its effects on gut-derived short-chain fatty acids (SCFAs). These metabolites modulate systemic immune responses, reduce circulating inflammatory mediators, and contribute to improved host–microbial homeostasis across distant mucosal sites, including the oral cavity [23].

**Figure 2:**
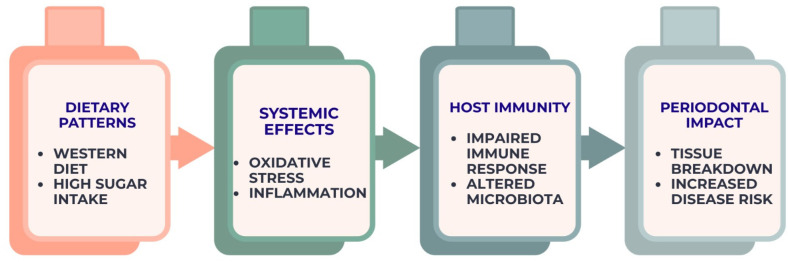
Diet–inflammation–immunity cascade contributing to periodontal breakdown. The figure represents an integrative conceptual framework derived from current observational, interventional, and mechanistic evidence.

**Figure 3:**
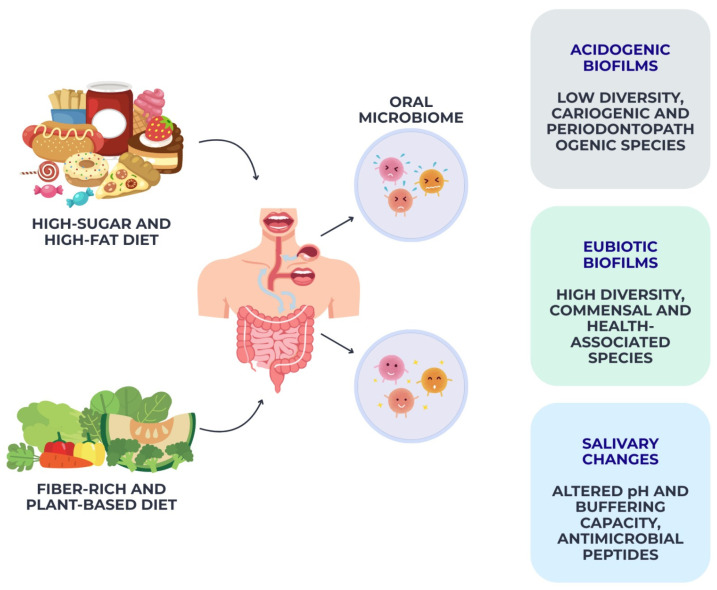
Diet-driven modulation of the oral microbiome. This schematic illustrates integrative framework synthesizing evidence from microbiome, nutritional, and experimental studies.

**Figure 4:**
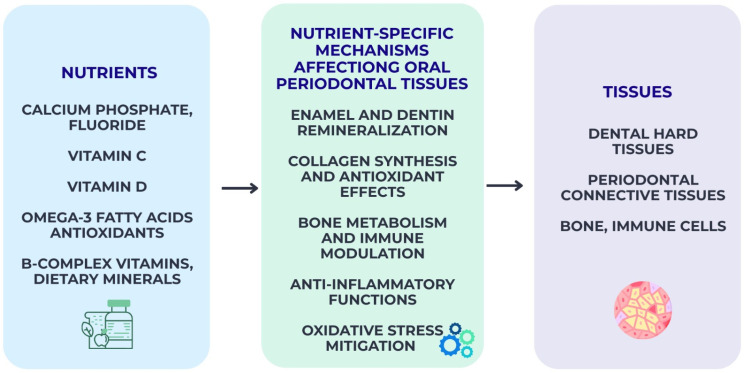
Nutrient-specific biological pathways influencing oral and periodontal tissues. The pathways shown represent an evidence-informed conceptual synthesis rather than a definitive causal model.

**Figure 5:**
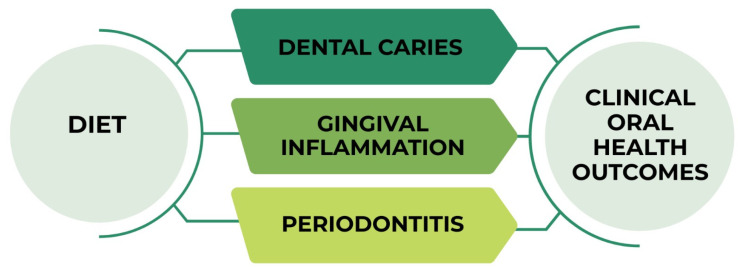
Clinical oral outcomes associated with dietary patterns. This figure summarizes integrative relationships reported across clinical and epidemiological studies.

The mechanistic pathways illustrated in Figure 2, Figure 3, Figure 4 and Figure 5 are intended as integrative conceptual frameworks that synthesize converging evidence from observational, interventional, and experimental studies. These schematic representations do not depict definitive causal models but rather summarize biologically plausible interactions between dietary exposures, systemic inflammation, microbiome modulation, and oral or periodontal outcomes, based on the current state of evidence.

Collectively, the evidence indicates that systemic physiological responses shaped by dietary exposures constitute a primary mechanistic link between nutrition and periodontal disease progression, a relationship schematically depicted in Figure 2.

### 2.3. Diet-Induced Modulation of the Oral Microbiome

Diet is one of the most powerful and immediate modulators of the oral microbiome, shaping its diversity, ecological stability, and pathogenic potential. High-sugar and high-refined-carbohydrate diets promote the selection of acidogenic and aciduric microorganisms such as *Streptococcus mutans*, *Lactobacillus* spp., and *Scardovia wiggsiae*, driving demineralization and biofilm maturation under low-pH conditions [24]. Frequent sugar exposure disrupts the ecological balance of the oral biofilm, reducing microbial diversity while favoring cariogenic niches capable of thriving in repeated acid challenges [25]. Similarly, diets rich in saturated fats and ultra-processed foods contribute to a pro-inflammatory microbial profile, increasing the abundance of proteolytic and periodontopathogenic species such as *Porphyromonas gingivalis*, *Fusobacterium nucleatum*, and *Tannerella forsythia*, thereby enhancing periodontal pathogenicity [26].

In contrast, fiber-rich, polyphenol-rich, and plant-forward diets support a more diverse and stable oral microbiome, promoting the growth of commensal species associated with oral health. Polyphenols derived from berries, green tea, and cocoa have been shown to inhibit the growth and virulence of several oral pathogens, regulate quorum sensing, and modulate biofilm architecture [27].

Dietary fiber, through its effects on systemic metabolism and salivary secretion, helps maintain a neutral pH environment and reduces the ecological advantage of aciduric species [28]. Additionally, omega-3 fatty acids and antioxidant-rich foods demonstrate selective antimicrobial and immunomodulatory effects, suppressing pathogenic biofilms while promoting eubiotic communities [29].

Diet-driven salivary changes also influence microbial ecology. High-sugar diets lower salivary pH, decrease buffer capacity, and alter mucin composition, conditions that support acidogenic bacteria [30]. In contrast, nutrient-dense diets stimulate salivary flow, enhance buffering capacity, and increase antimicrobial peptide activity—factors that create an unfavorable environment for oral pathogens [31].

In addition to periodontal disease, dietary patterns exert a well-established influence on dental caries development through distinct biological mechanisms. Frequent consumption of fermentable carbohydrates increases substrate availability for acidogenic and aciduric oral bacteria, resulting in prolonged acid exposure, enamel demineralization, and disruption of the demineralization–remineralization balance. Dietary habits also modulate salivary flow, buffering capacity, and mineral content, which are critical protective factors against caries progression. Moreover, micronutrient deficiencies—particularly calcium, phosphate, vitamin D, and vitamin C—may impair enamel integrity and reparative processes, further increasing caries susceptibility. Together, these mechanisms underscore the relevance of diet not only for periodontal inflammation but also for caries as a major oral health outcome.

Collectively, these findings demonstrate that habitual dietary patterns exert both direct and indirect effects on oral microbiome composition, driving shifts toward either a health-associated eubiotic community or a dysbiotic, disease-prone ecosystem, as schematically illustrated in Figure 3.

### 2.4. Nutrient-Specific Mechanisms Affecting Oral and Periodontal Tissues

Specific nutrients exert targeted biological effects on oral and periodontal tissues, influencing mineral balance, collagen stability, immune response, and microbial ecology. Calcium, phosphate, and fluoride form the cornerstone of enamel and dentin homeostasis, facilitating remineralization and enhancing resistance to acid challenges generated during carbohydrate fermentation. Adequate calcium–phosphate availability supports hydroxyapatite stability. In addition, fluoride incorporation into dental hard tissues lowers critical pH levels and inhibits bacterial glycolysis, thereby reducing caries risk [32].

Vitamin C plays a central role in periodontal connective tissue integrity through its involvement in collagen synthesis and fibroblast metabolism. Deficiency leads to impaired collagen maturation, increased oxidative stress, and heightened gingival bleeding, even in the absence of microbial overload [33]. Similarly, vitamin D contributes to periodontal health by regulating bone mineral metabolism, enhancing innate immunity through antimicrobial peptide expression, and modulating inflammatory pathways. Low serum levels are consistently associated with deeper periodontal pockets and a reduced therapeutic response [34].

Omega-3 polyunsaturated fatty acids, such as EPA and DHA, exert potent anti-inflammatory effects by serving as precursors for specialized pro-resolving mediators (SPMs). These mediators actively downregulate excessive neutrophil activity and support the resolution of inflammation. Clinical evidence suggests supplemental omega-3 improves periodontal parameters when combined with conventional therapy [35]. Antioxidants—particularly polyphenols, carotenoids, and vitamin E—counteract oxidative stress common in periodontal inflammation, inhibit bacterial virulence factors, and support tissue repair mechanisms [36].

B-complex vitamins and dietary minerals also contribute to oral and periodontal physiology. Folate supports epithelial turnover, while magnesium and zinc participate in enzymatic pathways essential for bone metabolism and immune function. Insufficient intake of these micronutrients has been linked to compromised periodontal healing and altered host responses [37,38,39].

To provide a clearer overview of the major dietary exposures, underlying biological pathways, and their implications for oral and periodontal health, the key mechanisms identified in the reviewed literature are summarized in Table 1 and Table 2.

This integrative synthesis highlights how specific nutrients, dietary components, and broader eating patterns modulate inflammatory responses, microbiome composition, and host tissue physiology, ultimately influencing the development and progression of dental caries, gingival inflammation, and periodontal disease.

Nutrient-specific effects underscore the mechanistic basis through which diet influences susceptibility to oral disease, complementing broader microbiome- and inflammation-related pathways illustrated in Figure 4.

### 2.5. Diet and Clinical Oral Health Outcomes

Dietary patterns have a measurable impact on the clinical expression of oral diseases, particularly dental caries, gingival inflammation, and periodontitis [40]. The most consistent clinical evidence concerns the role of free sugars in caries development, where intake frequency and total daily exposure are directly linked to lesion initiation and progression. Repeated sugar challenges lower plaque pH and favor acidogenic biofilms, resulting in enamel demineralization and increased DMFT indices across age groups [41]. Epidemiological data confirms a dose–response relationship in which individuals with high sugar intake exhibit significantly higher caries prevalence compared with those following low-sugar diets, independent of oral hygiene practices [42].

Beyond caries, dietary composition also influences gingival inflammation. Diets rich in saturated fats and ultra-processed foods are associated with elevated systemic inflammation and increased gingival bleeding scores, while anti-inflammatory dietary patterns have been linked to reduced gingivitis severity and improved periodontal indices [43]. Randomized trials demonstrate that short-term transition to a diet rich in omega-3 fatty acids, antioxidants, and fiber can reduce gingival inflammation even without changes in plaque accumulation. These findings suggest a host-modulating effect that is independent of mechanical biofilm removal [44].

Periodontitis outcomes also correlate with dietary exposures. Low intake of vitamin C, vitamin D, calcium, and omega-3 fatty acids is associated with deeper periodontal pockets, increased attachment loss, and reduced success of periodontal therapy [45]. Conversely, adherence to dietary patterns such as the Mediterranean diet has been linked with lower periodontitis prevalence, improved probing depths, and reduced inflammatory markers (e.g., IL-1β, CRP) [46]. Diet-induced changes in salivary flow and buffering capacity further modulate clinical risk, as reduced salivary protection in dehydrating or acidic diets increases vulnerability to both soft- and hard-tissue pathology [47]. Dietary behavior is a significant and modifiable determinant of oral disease expression, complementing microbial and host-mediated mechanisms, as illustrated in Figure 5.

### 2.6. Effects of Dietary Patterns on Oral and Periodontal Health

Dietary patterns, as opposed to individual nutrients, offer a more comprehensive view of habitual eating behavior and its cumulative impact on oral and periodontal health. The Western diet refers to a dietary pattern typically characterized by high consumption of refined carbohydrates, added sugars, saturated and trans fats, red and processed meats, and ultra-processed foods, alongside a low intake of fruits, vegetables, whole grains, and dietary fiber. This pattern is commonly associated with increased inflammatory and metabolic burden and has been frequently examined in relation to chronic disease risk. It has consistently been associated with increased systemic inflammation, higher caries prevalence, and more severe periodontal disease [48]. Individuals adhering to Western-style diets demonstrate elevated levels of inflammatory markers such as IL-6 and CRP, increased plaque accumulation, and deeper periodontal pockets, independent of mechanical oral hygiene practices [49]. The combination of frequent sugar exposure and pro-inflammatory nutrient profiles amplifies both microbial dysbiosis and host susceptibility, creating an environment conducive to both caries and periodontitis [50].

In contrast, the Mediterranean diet, which emphasizes fruits, vegetables, whole grains, legumes, nuts, olive oil, fish, and moderate wine intake, exerts protective effects on oral tissues [51]. This pattern is rich in polyphenols, antioxidants, omega-3 fatty acids, and fiber—all of which contribute to reduced gingival inflammation, improved periodontal parameters, and lower caries risk [52]. Observational and interventional studies have shown that adherence to a Mediterranean diet correlates with reduced probing depths, decreased bleeding on probing, and improved periodontal healing following therapy [53,54]. The anti-inflammatory and antioxidant properties of this dietary pattern appear to modulate both microbial ecology and host immune responses.

Plant-based diets offer varied oral health outcomes depending on their composition. Diets high in whole plant foods generally support increased microbial diversity and lower inflammatory burden; however, certain vegan or fruit-heavy diets may increase exposure to dietary acids, lowering salivary pH and potentially increasing enamel erosion risk [55].

A plant-based diet refers to a dietary pattern that emphasizes foods of plant origin, including fruits, vegetables, legumes, whole grains, nuts, and seeds, while limiting the intake of animal-derived products. Such dietary patterns vary in composition and are generally characterized by high fiber content and a diverse profile of bioactive compounds. Conversely, low-carbohydrate and ketogenic dietary patterns may reduce cariogenic risk due to decreased glucose availability but can also cause changes in salivary composition and microbiome profiles that require further investigation [53].

Anti-inflammatory and whole-food diets, including DASH-like patterns, have been associated with lower gingival inflammation and improved periodontal outcomes, even when controlling confounders such as smoking or oral hygiene [56,57]. These dietary frameworks collectively demonstrate that oral health is shaped not only by specific nutrients but by the broader dietary context in which they are consumed. Understanding these patterns provides clinically relevant insight into preventive strategies that integrate nutritional counseling into routine dental care [58]. Within this body of evidence, a limited number of well-designed dietary intervention trials stand out as key references, providing particularly informative data on the relationship between nutritional exposures, inflammatory modulation, and periodontal outcome [59,60,61,62].

### 2.7. Nutrition in Dental Prevention and Clinical Management

Nutrition plays a critical but often underutilized role in routine dental prevention and clinical management of oral and periodontal diseases. Dietary counseling is increasingly recognized as an essential component of preventive dentistry, as eating behaviors directly influence risk, gingival inflammation, and periodontal stability. Evidence indicates that targeted nutritional interventions—particularly reductions in free-sugar intake, increased consumption of fiber and antioxidant-rich foods, and improved vitamin D status—can substantially reduce caries incidence and support periodontal healing [63]. Despite its clinical relevance, nutritional guidance remains inconsistently implemented, with many patients receiving minimal or no dietary assessment as part of standard dental care [64].

Integrating nutrition into periodontal therapy has shown promising results. adjunctive anti-inflammatory diets, including those rich in omega-3 polyunsaturated fatty acids, polyphenols, and whole-food plant-based components, have been associated with reductions in bleeding on probing, probing depth, and inflammatory biomarkers, even when plaque levels remain unchanged [65,66,67,68].

Similarly, low-glycemic and low-sugar dietary modifications enhance glycemic control in patients with diabetes, resulting in improved periodontal outcomes and decreased disease progression [69]. These findings suggest that modifying systemic inflammatory load through diet can act synergistically with mechanical biofilm removal [70].

In groups at elevated risks such as older adults, individuals with xerostomia, patients with diabetes, smokers, and those with low socioeconomic status—nutritional assessment becomes even more relevant [71]. Reduced salivary flow, impaired mastication, and limited access to nutrient-dense foods can exacerbate oral disease susceptibility, making personalized dietary strategies crucial for disease prevention and maintenance [72,73]. Additionally, emerging clinical frameworks advocate interdisciplinary collaboration between dentists, nutritionists, and medical professionals to address diet-related oral conditions more effectively [74].

To provide an overview of the studies selected for inclusion and to indicate where they are discussed within the manuscript, Table 3 summarizes the articles cited across the main thematic items of this narrative review.

Despite the potential relevance of nutritional counseling in preventive dentistry, several practical barriers currently limit its routine implementation. Time constraints during dental appointments, limited formal training in nutrition among dental professionals, and variable patient adherence to dietary recommendations represent important challenges. In addition, the lack of standardized, validated, and time-efficient dietary assessment tools tailored for dental settings complicates consistent integration into clinical workflows. These limitations highlight the need for pragmatic strategies, targeted education, and the development of simple, standardized dietary screening instruments to support evidence-informed nutritional counseling in dental practice.

From a clinical perspective, integrating nutritional counseling into dental practice does not necessarily require extensive dietary interventions but can be achieved through targeted, risk-based strategies. Brief dietary screening tools, focused on sugar intake, ultra-processed food consumption, and micronutrient adequacy, may be incorporated into routine dental assessments alongside traditional risk evaluation. Dentists and dental hygienists can provide concise, evidence-based nutritional guidance aimed at reducing cariogenic exposure, supporting anti-inflammatory dietary patterns, and reinforcing periodontal stability. In patients with increased systemic or periodontal risks such as those with diabetes, xerostomia, or recurrent periodontal inflammation—interdisciplinary collaboration with nutritionists or primary care providers may further enhance preventive and therapeutic outcomes. Such integrative approaches position nutrition as a complementary component of comprehensive oral health management.

It is important to interpret the associations described in this review in light of the type of evidence from which they originate. Observational studies consistently report associations between dietary patterns, nutrient intake, and oral or periodontal outcomes; however, these designs cannot establish causality due to potential confounding and reverse causation. Interventional studies, although fewer in number and often limited by short duration or combined therapeutic approaches, provide stronger evidence supporting a causal role of specific dietary modifications in modulating periodontal inflammation and clinical parameters. Mechanistic and experimental studies further contribute biological plausibility by elucidating inflammatory, microbial, and metabolic pathways through which diet may influence oral tissues. Together, these complementary lines of evidence support a causal framework, while also underscoring the need for well-designed long-term dietary intervention trials.

## 3. Future Perspectives

The present narrative review is subject to several methodological considerations that should be acknowledged when interpreting its findings. Because the review does not follow a systematic design, it does not include a formal risk-of-bias assessment or quantitative synthesis of study outcomes, which may limit the comparability of results across heterogeneous study designs. It synthesized current evidence linking dietary factors with oral and periodontal health, integrating findings from observational, interventional, and mechanistic studies. The reviewed literature highlights consistent associations between dietary patterns, inflammatory modulation, and microbiome-related pathways relevant to periodontal outcomes, while also underscoring the heterogeneity of study designs and assessment methods. By bringing together nutritional, inflammatory, and microbiome-related perspectives, this review provides a structured overview of the current state of evidence and identifies key areas where knowledge remains incomplete.

Although periodontal disease is emphasized due to the depth and consistency of available mechanistic evidence, the reviewed literature also suggests that dietary patterns may influence dental caries risk and broader oral health outcomes. These associations, while supported by biological plausibility and observational data, warrant further investigation to clarify their magnitude and clinical implications.

Despite the inherent limitations of a narrative approach, this review presents several strengths. It integrates evidence across microbiome research, inflammatory pathways, and nutritional science, providing a multidimensional perspective on oral and periodontal health. The synthesis is based on up-to-date clinical, epidemiological, and mechanistic literature, reflecting current research directions in the field. In addition, the review emphasizes clinical relevance by linking biological mechanisms with dietary patterns and practical implications for dental care. The structured thematic organization further facilitates interpretation and supports the translation of evidence into preventive and therapeutic contexts.

Dietary assessment methods varied substantially between studies, ranging from self-reported food frequency questionnaires to objective biomarker analyses, introducing potential measurement inconsistencies and recall bias. Similarly, differences in periodontal diagnostic criteria, microbiome sequencing techniques, and definitions of caries endpoints contribute to variation in reported outcomes and limit cross-study uniformity.

Publication bias remains another inherent limitation, as studies reporting significant findings are more likely to be published, potentially skewing the available evidence. Although efforts were made to include a comprehensive range of clinical, epidemiological, and mechanistic research, the synthesis is ultimately constrained by the quality and completeness of the original studies. Nonetheless, the integration of diverse evidence sources provides a broad and informative overview of the diet–oral health relationship, offering valuable insights despite these inherent limitations.

Despite growing evidence linking dietary patterns, nutrient intake, and oral and periodontal health, several important gaps and inconsistencies limit the strength of current conclusions. The most significant challenge is the heterogeneity of dietary assessment methods. Many studies rely on self-reported dietary intake such as food-frequency questionnaires or 24 h recalls—which are prone to recall bias and do not reliably capture habitual patterns or the complexity of mixed meals [75,76,77,78,79]. Similarly, considerable variation exists in the diagnostic criteria for periodontal disease, the indices used to measure caries activity, and the methodologies for profiling the oral microbiome. These inconsistencies complicate cross-study comparisons and weaken the capacity to draw unified mechanistic models.

Interventional evidence also remains limited. While observational studies consistently link Western-style diets to poorer oral outcomes and Mediterranean or whole-food diets to improved periodontal status, randomized controlled trials investigating diet as a primary intervention remain scarce. Most existing trials are short-term, small in sample size, and often combine dietary changes with mechanical debridement, making it difficult to isolate the independent effect of nutrition [46,80]. High-quality, long-term trials are needed to determine the causal impact of specific dietary modifications on caries incidence, gingival inflammation, and periodontal disease progression [81].

Another major gap concerns the interaction between diet, the oral microbiome, and systemic health. Although mechanistic evidence suggests that dietary components shape microbial composition and influence host immunity, integrating these findings into predictive clinical models remains challenging.

Multi-omics approaches—incorporating microbiome sequencing, metabolomics, salivary proteomics, and inflammatory biomarkers—are emerging but currently underused in oral research [82,83]. Their application in large, diverse population cohorts would significantly advance understanding of how dietary exposures translate into measurable oral health outcomes.

Vulnerable populations also require deeper investigation. Older adults, individuals with systemic diseases such as diabetes, those with limited access to nutritious foods, and patients with xerostomia or masticatory difficulties are disproportionately affected by diet-related oral conditions but remain underrepresented in current studies [57]. Future research should prioritize these groups to improve equity in preventive and therapeutic strategies.

The interpretation of findings presented in this narrative review should consider several factors influencing the strength and reliability of the available evidence. The included studies comprise a heterogeneous mix of observational, interventional, and mechanistic research, each associated with distinct sources of bias and methodological constraints. Observational studies are particularly susceptible to residual confounding, measurement error in dietary assessment, and reverse causation, while interventional trials often vary in duration, sample size, and intervention complexity.

## 4. Conclusions

This narrative review highlights the central role of diet as a determinant of oral and periodontal health, acting through interconnected metabolic, inflammatory, microbial, and nutrient-dependent pathways. Diets rich in refined carbohydrates, saturated fats, and ultra-processed foods promote systemic inflammation, oxidative stress, and oral microbiome dysbiosis, thereby increasing susceptibility to dental caries, gingivitis, and periodontitis.

In contrast, nutrient-dense and anti-inflammatory dietary patterns, such as the Mediterranean diet, plant-forward diets, and whole-food frameworks—support a more balanced host immune response, enhance microbial eubiosis, and improve clinical periodontal parameters. Micronutrients including vitamin C, vitamin D, calcium, magnesium, and omega-3 fatty acids exert essential roles in tissue repair, collagen metabolism, bone turnover, innate immunity, and inflammatory resolution.

While periodontal disease is emphasized due to its complex inflammatory and microbiome-mediated mechanisms, the findings also support diet as a relevant determinant of caries risk and overall oral health.

Beyond its conceptual relevance, the evidence synthesized in this narrative review has direct implications for clinical dental practice. The integration of nutritional considerations into routine oral health assessment may support preventive strategies, complement conventional periodontal therapy, and contribute to long-term disease management. By highlighting the interconnected roles of diet, systemic inflammation, and the oral microbiome, this review emphasizes the potential value of nutrition-informed approaches in enhancing personalized dental care.

From a practical standpoint, incorporating brief dietary screening and targeted nutritional guidance into dental settings may help identify patients at increased risk for periodontal disease progression or impaired therapeutic response. While further high-quality intervention studies are required to refine clinical recommendations, the current body of evidence supports the inclusion of nutrition as a relevant component of comprehensive oral health management. These insights reinforce the importance of interdisciplinary collaboration and provide a foundation for translating nutritional research into clinically meaningful and patient-centered dental care.

Although heterogeneity exists across study designs and dietary assessment tools, the overall evidence consistently indicates that nutritional habits are a modifiable and clinically significant component of oral disease prevention and management.

From a practical perspective, concise dietary screening in dental settings may prioritize the identification of excessive free sugar and ultra-processed food intake, alongside suboptimal vitamin D and omega-3 fatty acid status. Addressing these modifiable factors through targeted nutritional guidance may complement conventional periodontal therapy and support improved inflammatory control and treatment response. In this context, even brief, diet-focused interventions integrated into routine dental visits may help clinicians identify patients at higher risk of disease progression and facilitate more personalized, prevention-oriented oral healthcare strategies.

Integrating dietary counseling into routine dental care, alongside mechanical plaque control and risk-based therapy, may enhance periodontal stability and reduce the burden of diet-related oral conditions. Strengthening interdisciplinary collaboration between dentistry, nutrition, and general medicine is essential to translating these findings into personalized, sustainable dietary strategies that support long-term oral and systemic health.

## Figures and Tables

**Table 1:** Nutrients and food components associated with oral and periodontal outcomes.

Dietary Factor	Biological Pathway	Oral/Periodontal Effects	Key Evidence	References
Omega-3 (EPA/DHA)	SPMs → resolution of inflammation	Improved periodontal healing	Adjunct omega-3 improves BOP and PPD	[8,9,10,11,15,16]
Vitamin C	Collagen synthesis; antioxidant defense	Reduced bleeding, improved tissue repair	Deficiency → gingival bleeding	[21,33]
Vitamin D	Innate immunity; bone metabolism	Reduced periodontal inflammation	Low Vit D = deeper pockets	[22,34,35]
Polyphenols	Antioxidant; modulates microbiome	Reduced pathogen virulence; stable biofilm	Inhibits *P. gingivalis* and quorum sensing	[20,21,27,36]
Dietary fiber	SCFAs → immune modulation	Lower inflammation; healthier microbiome	Fiber ↑ microbial diversity	[20,23,28]

↑—increased.

**Table 2:** Dietary patterns associated with oral and periodontal outcomes.

Dietary Factor	Biological Pathway	Oral/Periodontal Effects	Key Evidence	References
High sugar/refined carbohydrates	Acidogenic shift; inflammatory cytokines ↑	Caries risk ↑; gingival inflammation	Frequent sugar lowers pH, promotes *S. mutans*	[17,18,38,39]
Saturated fats/ultra-processed foods	Inflammation ↑ (IL-6, TNF-α, CRP); oxidative stress	Periodontal breakdown; deeper pockets	Western diet increases inflammatory burden	[17,18,25,26]
Mediterranean diet	Anti-inflammatory; antioxidant-rich	Improved periodontal clinical outcomes	Lower CRP, improved PPD	[12,13,14]

↑—increased.

**Table 3:** Representative studies discussed across the main thematic sections of the review.

Review Item/Section	Type of Evidence	Representative Studies	Main Focus
Diet and Oral Health	Observational	[12,18,24]	Dietary patterns and oral health outcomes
Nutrition and Periodontal Inflammation	Interventional	[31,35,42]	Inflammatory modulation
Oral Microbiome and Diet	Mechanistic	[46,50,53]	Microbiome composition and function
Dietary Interventions	Interventional	[58,61,66]	Periodontal clinical parameters
Integrative Pathways	Mixed	[70,74,75,76,77,78,79]	Diet–inflammation–microbiome links

## Data Availability

No new data were created or analyzed in this study. Data supporting the findings of this review are available within the cited literature. The complete list of identified publications is available upon request from the authors.

## Abbreviations

The following abbreviations are used in this manuscript:

DASH diet	Dietary Approaches to Stop Hypertension
IL-6	Interleukin-6
TNF-α	Tumor Necrosis Factor α
SPMs	Specialized Pro-Resolving Mediators
SCFAs	Short-Chain Fatty Acids
BOP	Bleeding on Probing
PPD	Probing Pocket Depth
EPA	Eicosapentaenoic Acid
DHA	Docosahexaenoic Acid
DMFT	Decayed, Missing, Filled Teeth
